# Optimization of ^177^Lu-labelling of DOTA-TOC, PSMA-I&T and FAPI-46 for clinical application

**DOI:** 10.1186/s41181-023-00196-1

**Published:** 2023-05-26

**Authors:** Aylin Cankaya, Matthias Balzer, Holger Amthauer, Winfried Brenner, Sarah Spreckelmeyer

**Affiliations:** Charité – Universitätsmedizin Berlin, corporate member of Freie Universität Berlin, Humboldt-Universität zu Berlin, and Berlin Institute of Health, Department of Nuclear Medicine, Augustenburger Platz 1, 13353 Berlin, Germany

**Keywords:** Radiopharmaceuticals, Precursor load, Lutetium-177, Automatization

## Abstract

**Background:**

^177^Lu-radiopharmaceuticals are routinely used for the treatment of various tumor entities. The productions of radiopharmaceuticals follow strict good-manufacturing practice guidelines and synthesis optimizations thereof have a strong impact on e.g. the quality of the product, radiation safety and costs. The purpose of this study is to optimize the precursor load of three radiopharmaceuticals. For that, different precursor loads were evaluated and compared to previously reported findings.

**Results:**

All three radiopharmaceuticals were successfully synthesized in high radiochemical purities and yields on the ML Eazy. The precursor load was optimized for [^177^Lu]Lu-FAPI-46 from 27.0 to 9.7 µg/GBq, for [^177^Lu]Lu-DOTATOC from 11 to 10 µg/GBq and for [^177^Lu]Lu-PSMA-I&T from 16.3 to 11.6 µg/GBq.

**Conclusions:**

We successfully reduced the precursor load for all three radiopharmaceuticals while maintaining their quality.

**Supplementary Information:**

The online version contains supplementary material available at 10.1186/s41181-023-00196-1.

## Background

**Fig. 1:**
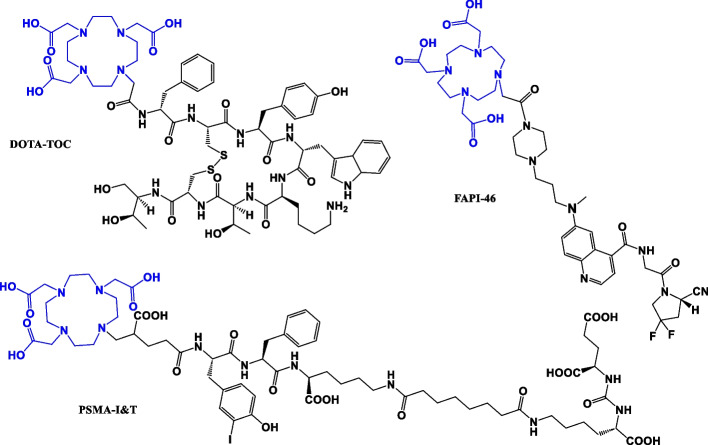
Chemical structures of the precursors investigated in this work

The clinical use of ^177^Lu-radiopharmaceuticals has increased in the past years, and two therapeutic radiopharmaceuticals received approval by both the FDA and EMA in the last five years (Lutathera and Pluvicto) (Harris and Zhernosekov 2022; Jia et al. 2022; Chakravarty and Chakraborty 2021; Hennrich and Eder 2022). Noteworthy, the preparation of in-house therapeutic radiopharmaceuticals is still an essential task of radiopharmacists/radiochemists in radiopharmacy facilities. Depending on the available equipment, syntheses are carried out either manually, (semi)-automated or fully automated, similarly as with ^68^Ga-radiopharmaceuticals (Nelson et al. 2022). Lutetium-177 is a medium-energy β-emitter, with 6.73 days of physical half-life. The emitted electrons (78.6%) show a maximum β-energy of 498 keV and penetrate surrounding tissue up to 2 mm. Lutetium-177 also shows two additional γ-emissions (11% and 6.4%) of 208 and 113 keV, respectively. To keep the dose for the production personnel as low as possible and the synthesis level as reproducible as possible, automated synthesis modules are used for routinely produced ^177^Lu-radiopharmaceuticals. Beside DOTA-TOC and PSMA-analogues, FAP analogues also received attention as theranostic radiopharmaceuticals (Fig. 1) (Treglia et al. 2021). Recently, FAPI-46 was successfully radiolabeled with lutetium-177 by Eryilmaz and Kilbas et al. (2021).

**Fig. 2:**
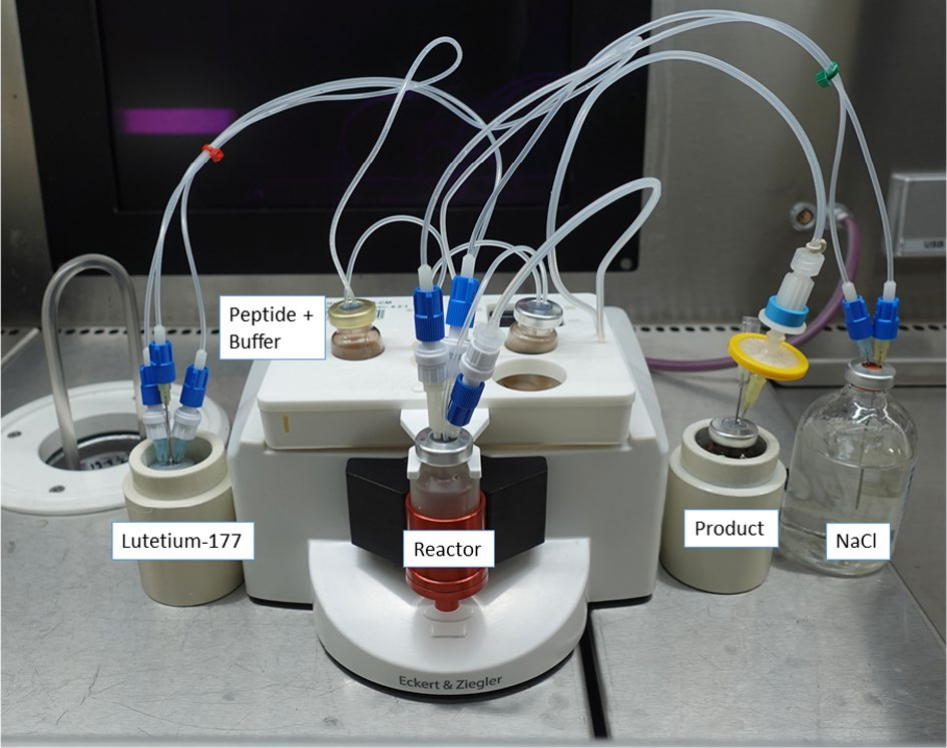
Set-up of cassette and ML eazy

To our knowledge, the synthesis of routinely used ^177^Lu-radiopharmaceuticals is usually performed with one patient dose of about 7.4 GBq lutetium-177. Here, we present a method for the automated synthesis of single batches of the three radiopharmaceuticals [^177^Lu]Lu-DOTA-TOC, [^177^Lu]Lu- PSMA-I&T and [^177^Lu]Lu-FAPI-46 using a total starting activity up to 26 GBq of [^177^Lu]LuCl_3_ for each batch, which is sufficient to obtain doses for treating up to three patients. The general setup of the module used can be seen in Fig. 2.

## Results

### [^177^Lu]Lu-DOTA-TOC synthesis for 1–3 patient doses

For the synthesis of [^177^Lu]Lu-DOTA-TOC, we used four times a typical lutetium-177 starting activity for single dose (5.8–8.6 GBq) and one batch for three doses (20.3 GBq). The amount of peptide was 115 µg/single dose (15.4–16.7 µg/GBq, synthesis no. 1–3) and 345 µg/batch of three doses (17.0 µg/GBq, synthesis no. 4). For the 5th reaction, we used 58 µg/single dose (10 µg/GBq) (Table 1).

**Table 1:** Results of [^177^Lu]Lu-DOTA-TOC

Synthesis no	Starting activity (MBq)	Amount of DOTA-TOC (µg/GBq)	Balanced radiochem. yield (%)	Radiochem. purity (%) radio-HPLC	Radiochem. purity (%) TLC
1	7478	15.4	92	99.8	99.9
2	8485	16.7	89	99.7	99.9
3	8561	16.4	91	99.6	99.9
4	20,270	17.0	98	99.6	99.8
5	5806	10.0	97	99.9	99.2

All syntheses were successful. Particularly noteworthy, the fourth reaction with a starting activity of approx. 20 GBq yielded to similar results compared to a single dose synthesis. In the clinical routine, we could confirm, that with similar higher starting activities of 22.9 GBq (15.0 µg/GBq DOTA-TOC) and 24.8 GBq (13.9 µg/GBq DOTA-TOC), we achieved > 99% radiochemical purity. All quality control parameters were within standard specifications (Supporting information). In comparison to the synthesis described by Iori et al. (2017), we achieved an improved balanced radiochemical yield of 93.4 ± 3.9% with a decreased precursor load of 10–17 µg (Table 2). As shown in the Additional file 1: Figure S1, radiolytic side-products in the range of 0.1% were detectable when using 20.3 GBq Lu-177.

**Table 2:** Comparison of reaction conditions of Charité (synthesis no.1–5) with Iori et al.

	Charité	Iori et al. (Iori et al. 2017)
Reaction temperature (°C)	95	90
Reaction time (min)	30	30
RCY (%)	93.4 ± 3.9	83.1 ± 5.7
RCP (%) radio-HPLC	99.7 ± 0.2	99.3
RCP (%) TLC	99.7 ± 0.3	98.4
amount of DOTA-TOC (µg/GBq)	10.0–17.0	11

### [^177^Lu]Lu-PSMA-I&T synthesis for 1–3 patient doses

For the synthesis of [^177^Lu]Lu-PSMA-I&T (Table 3), we used in analogy to [^177^Lu]Lu-DOTA-TOC three times a typical lutetium-177 dose for one patient (6.1 GBq- 6.8 GBq, synthesis no 1–3) and one in a batch for three patients (25.9 GBq, synthesis no. 4). In the first synthesis, a noteworthy amount of activity/product was lost on the CM cartridge and the sterile filter, that lead to a low balanced radiochemical yield of 78% vs. 94% in the second synthesis The CM cartridge is a cation exchanger, that holds back unbound lutetium-177. After the first synthesis was finished, we eluted the CM cartridge with additional 5 mL of 0.9% NaCl solution in order to distinguish between bound and unbound lutetium-177 on the CM cartridge. Unbound lutetium-177 would stay on the CM cartridge and bound lutetium-177 (e.g. product) would elute with the 0.9% NaCl solution. The eluted solution contains product as analyzed via radio-HPLC and the remaining activity on the CM cartridge was neglectable. By increasing the amount of 0.9% NaCl solution from 5 to 10 mL for rinsing the reaction vessel, we increased the balanced radiochemical yield from 78 to 96.3% ± 2.1% (synthesis no. 2–4). As shown in the Additional file 1: Fig. S2, radiolytic side-products of 0.8% appeared when using 25.9 GBq lutetium-177. In the clinical routine, we could confirm, that with similar higher starting activities of 22.4 GBq (13.4 µg/GBq PSMA-I&T) and 20.6 GBq (14.6 µg/GBq PSMA-I&T), we achieved > 99% radiochemical purity.

**Table 3:** Results of [^177^Lu]Lu-PSMA-I&T

Synthesis no	Starting activity (MBq)	Amount of PSMA-I&T (µg/GBq)	Balanced radiochem. yield (%)	Radiochem. purity (%) radio-HPLC	Radiochem. purity (%) TLC
1	6130	16.3	78	99.7	99.4
2	6836	14.6	94	99.7	99.9
3	6764	14.8	97	99.8	99.9
4	25,890	11.6	98	98.9	99.9

### [^177^Lu]Lu-FAPI-46

For establishing the synthesis of [^177^Lu]Lu-FAPI-46 (Tables 4, 5), we performed five reactions with starting activities ranging from 6.7 to 8.3 GBq. The load of FAPI-46 was kept constant for all reactions with 100 µg. In the first two syntheses, activity/product was lost on the CM cartridge and the sterile filter. By increasing the amount of NaCl solution from 5 to 10 mL for rinsing the reaction vessel, we increased the balanced radiochemical yield from 86.0% ± 1.4% (synthesis no 1 and 2) to 95% ± 1.0% (synthesis no. 3–5). Comparing our results to the synthesis parameters of Eryilmaz et al., we achieve a higher balanced radiochemical yield with less amount of FAPI-46, 11.9 µg/GBq vs. 27 µg/GBq. No radiolytic side-products were observed for the applied radioactivities (Additional file 1: Figure S3).

**Table 4:** Results of [^177^Lu]Lu-FAPI-46

Synthesis no	Starting activity (MBq)	Amount of FAPI-46 (µg/GBq)	Balanced radiochem. yield (%)	Radiochem. purity (%) radio-HPLC	Radiochem. purity (%) TLC
1	7914	12.6	85	99.8	99.8
2	7618	17.1	87	99.3	99.7
3	8279	9.7	95	99.8	100.0
4	8087	12.4	96	99.4	99.9
5	6749	11.9	94	99.0	99.9

**Table 5:** Comparison of reaction conditions of Charité (synthesis no.3) with Eryilmaz et al.

	Charité	Eryilmaz et al. (2021)
Reaction temperature (°C)	95	90
Reaction time (min)	30	20
RCY (%)	95	86
RCP (%) radio-HPLC	99.8	> 99
RCP (%) TLC	100.0	98.4
Amount of FAPI-46 (µg/GBq)	9.7	27

## Discussion

The preparations of ^177^Lu-radiopharmeuticals for up to three patient doses in one reaction setup have not been reported yet to our knowledge. The manufacturer of the disposable cassettes does only describe the use of 40 µg/GBq of the precursor per synthesis. If using the recommended amount of peptide, we would need to use 296 µg of precursor for a single dose (e.g. 7.4 GBq) and about 900 µg for a batch of three patient doses (e.g. 22.2 GBq) of DOTA-TOC or PSMA-I&T. For single-dose FAPI-46, we would need to use 268–332 µg of precursor. Of note, other radiopharmacies already adapted the precursor load used per synthesis on the ML eazy from 40 to 11 µg/GBq for DOTA-TOC (Iori et al. 2017) and 27 µg/GBq for FAPI-46 (Eryilmaz and Kilbas 2021). Extensive efforts by our radiopharmacy to further decrease the amount of precursor are limited due to the fact, that purchasing high activities of lutetium-177 for test syntheses is very expensive. In the frame of synthesis validation, we optimized the precursor load and succeeded to decrease the amount of DOTA-TOC to 10 µg/GBq, PSMA-I&T to 11.6–16.3 µg/GBq, and FAPI-46 to 9.7 µg/GBq while maintaining the high quality of the product. By this, the precursor load can be effectively adapted for each synthesis, which reduces the costs per synthesis. In addition, the costs can be further decreased by using one cassette for a batch synthesis for three patients instead of using three cassettes for single dose synthesis.

With respect to the fact that unlabeled precursor is not eliminated in the final product during standard synthesis approaches, the molar activity plays an important role. Consequently, decreasing the precursor load has two advantages: firstly, increasing the molar activity for the benefit of quality of application, and secondly, lowering the costs of synthesis. As discussed before by Hooijman et al. (2022), the appearance of radiolytic side-products have an impact on radiopharmaceutical binding to cells, e.g. of [^177^Lu]Lu-PSMA-I&T. To prevent this phenomenon, we recommend to dilute the product solution with sodium chloride 0.9% immediately after synthesis in order to decrease radiolysis.

## Conclusions

The syntheses of three different ^177^Lu-radiopharmeuticals were successfully optimized with regard to the respective precursor load/GBq lutetium-177.

## Methods

### Materials

DOTA-TOC (GMP quality, product no. 9702) was purchased from ABX, Radeberg, Germany and PSMA-I&T (GMP quality, product no. 000.000.CA0.1000.002R) from piCHEM, Raaba-Grambach, Austria. FAPI-46, manufactured by ABX, Radeberg, Germany, was made available free of costs from SOFIE Biosciences. All chemicals were of pure chemical grade, and solvents for high-pressure-liquid-chromatography (HPLC) were obtained as HPLC grade. TraceSelect water (Sigma-Aldrich Chemie GmbH, Taufkirchen, Germany) was used in all experiments. The pharmaceutical grade lutetium-177 (EndolucinBeta) for single patient doses was purchased from ITM Radiopharma, Garching/Munich, Germany, lutetium-177 n.c.a. 20.27 GBq from Monrol, Gebze/Kocaeli, Turkey, and lutetium-177 n.c.a. 25.89 GBq from Eckert&Ziegler Radiopharma GmbH, Braunschweig, Germany. ModularLab EAZY (Eckert & Ziegler Eurotope GmbH, Berlin, Germany) and the buffer ascorbic acid 50 mg with NaOH 7.9 mg (Polatom, Otwock, Poland) were used. Activity counting was performed using a borehole counter (Nuklear-Medizintechnik Dresden GmbH, Dresden, Germany). HPLC was performed using the HPLC system Knauer Azura (UVD: 2.1 L; P6.1L) coupled with UV and radiometric (Raytest Socket 2′′8103 0370) detectors. The column used is a Synergi™ 4 µm Fusion-RP 80Å, 250 × 4.6 mm. For TLC measurements, the TLC scanner MiniGita from Raytest and TLC Silica gel 60 RP-18 F_254_ (Sigma-Aldrich) were used. The test for endotoxins was performed using Nexgen PTS (Charles River). The CM cartridge is a cation-exchange cartridge from Sep-Pak Accell Plus light 130 mg sorbent per cartridge, 37–55 μm particle size. The sterility tests were carried out as required by the European Pharmacopoeia by the Institut für Hygiene, Charité.

### Preparation for labeling of DOTA-TOC, PSMA-I&T and FAPI-46 with lutetium-177 on ML eazy

First, the commercially available fully automated synthesis platform ML eazy was equipped with a disposable single-use cassette (C0-LUDOTAPSMA-CM for PSMA-I&T and FAPI-46 or C0-LUDOTAPEP-CM for DOTA-TOC). FAPI-46 was prepared in 100 μg aliquots from a stock solution (1 mg/mL) in TraceSelect water. 115 µg DOTA-TOC (lyophilized) was solubilized with 2.5 mL of buffer (single dose), 3 × 115 µg DOTA-TOC were solubilized with 2.5 mL buffer (batch of three doses) or 1.0 mg DOTA-TOC were solubilized in 1000 µL TraceSelect water and 58 µL were used for the 5th synthesis. PSMA-I&T was prepared in 100 µg aliquots from a stock solution (1 mg/mL) in TraceSelect water. The ascorbic acid buffer was prepared following the manufacturer´s protocol: The solid was dissolved in 2.5 mL TraceSelect water. 2.5 mL ascorbic acid buffer plus the respective precursor were added into the vial with the golden cap. An extra sterile vial with 10.0 mL of 0.9% NaCl was attached to the cassette with the green needle. The other steps were in accordance with the manufacturer´s manual.

### Labeling with lutetium-177

The synthesis was performed fully automated without any user interaction. [^177^Lu]LuCl_3_ obtained from ITM was added to the synthesis cassette by connecting the respective needle of the cassette with the 3 mL V-shaped vial from ITM.

The following parameters were used for the labeling step:

- DOTA-TOC: 30 min, 95 °C
- PSMA-I&T: 20 min, 90 °C
- FAPI-46, 30 min, 90 °C

The reaction mixture was cooled down by adding 10.0 mL 0.9% NaCl to the reaction mixture. The crude reaction solution was subsequently transferred through the CM cartridge and a sterile filter into the product vial. A sample for quality control was taken. The radiochemical yield is expressed as “balanced radiochemical yield”, which means that the radioactivity of the different parts of the synthesis cassette (product vial, waste vial, reactor vial, C18 cartridge, sterility filter and SCX cartridge) were measured and the sum of all parts was taken as total radioactivity. The radiochemical yield of the synthesis was then expressed as ratio in % of the product vial divided by the sum.

### Quality control

After synthesis, the product vial was removed from the ML eazy module and further evaluated for quality control determining the following parameters: total product activity, chemical purity, pH, sterility, endotoxins and radiochemical purity (high-pressure-liquid chromatography and TLC). Radio-HPLC was performed with the following method. A: acetonitrile + 0.1% TFA; B: water + 0.1% TFA, gradient: 0–8 min A: 0–50%; 8–10 min A: 50%; 10–12 min A: 50–0% with a flow-rate of 1 mL/min. For iTLC, citrate buffer 0.05 M at pH 5 was used as mobile phases and silicagel strips as stationary phase. The sterility tests were carried out as described in the European Pharmacopoeia by the Institut für Hygiene, Charité.

## Supplementary Information

**Additional file 1.** Supplementary information.

## Data Availability

All data generated or analyzed during this study are included in this published article.

## Abbreviations

FAP: Fibroblast activation protein
FAPI: Fibroblast activation protein inhibitor
n.d.c.: Non decay corrected
RCY: Radiochemical yield
MLPT: Modular Lab PharmTracer
ML eazy: Modular Lab eazy
PET: Positron Emission Tomography
HPLC: High-pressure-liquid-chromatography
TFA: Trifluoro acetic acid
