# Supplementary material for: Optimization of 177Lu-labelling of DOTA-TOC, PSMA-I&T and FAPI-46 for clinical application
Source: EJNMMI Radiopharm Chem. 2023 May 26;8:10. doi: 10.1186/s41181-023-00196-1 (PMC10219910; doi:10.1186/s41181-023-00196-1)
Supplement: Supplementary file 1 — Additional file 1. Supplementary information. [file 41181_2023_196_MOESM1_ESM.docx]

Supplementary Information

**Optimization of [^177^Lu]-labeling of DOTA-TOC, PSMA-I&T and FAPI-46 for clinical application**

Authors: Aylin Cankaya,^1^ Matthias Balzer,^1^ Holger Amthauer,^1^ Winfried Brenner,^1^ Sarah Spreckelmeyer^1,*^


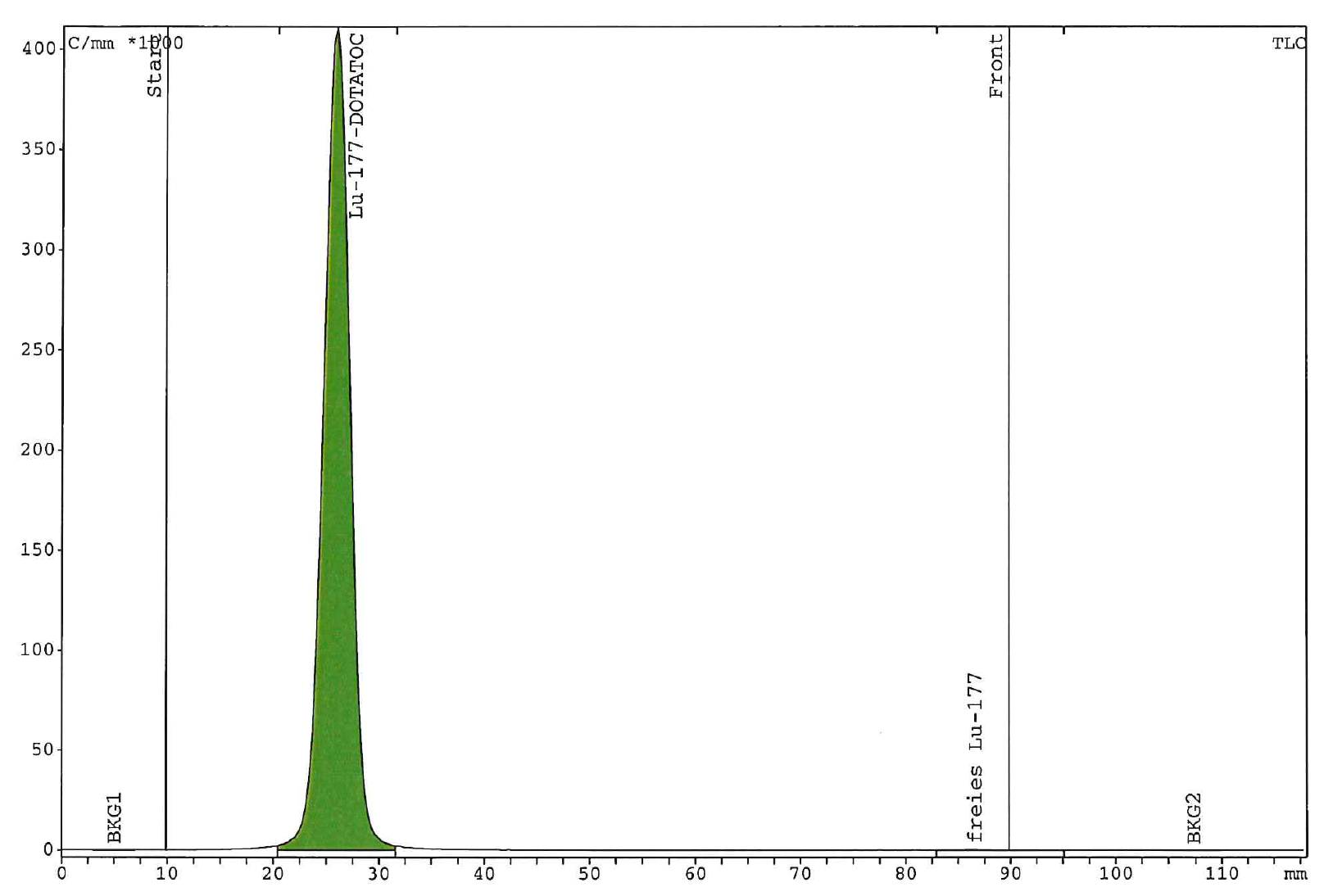

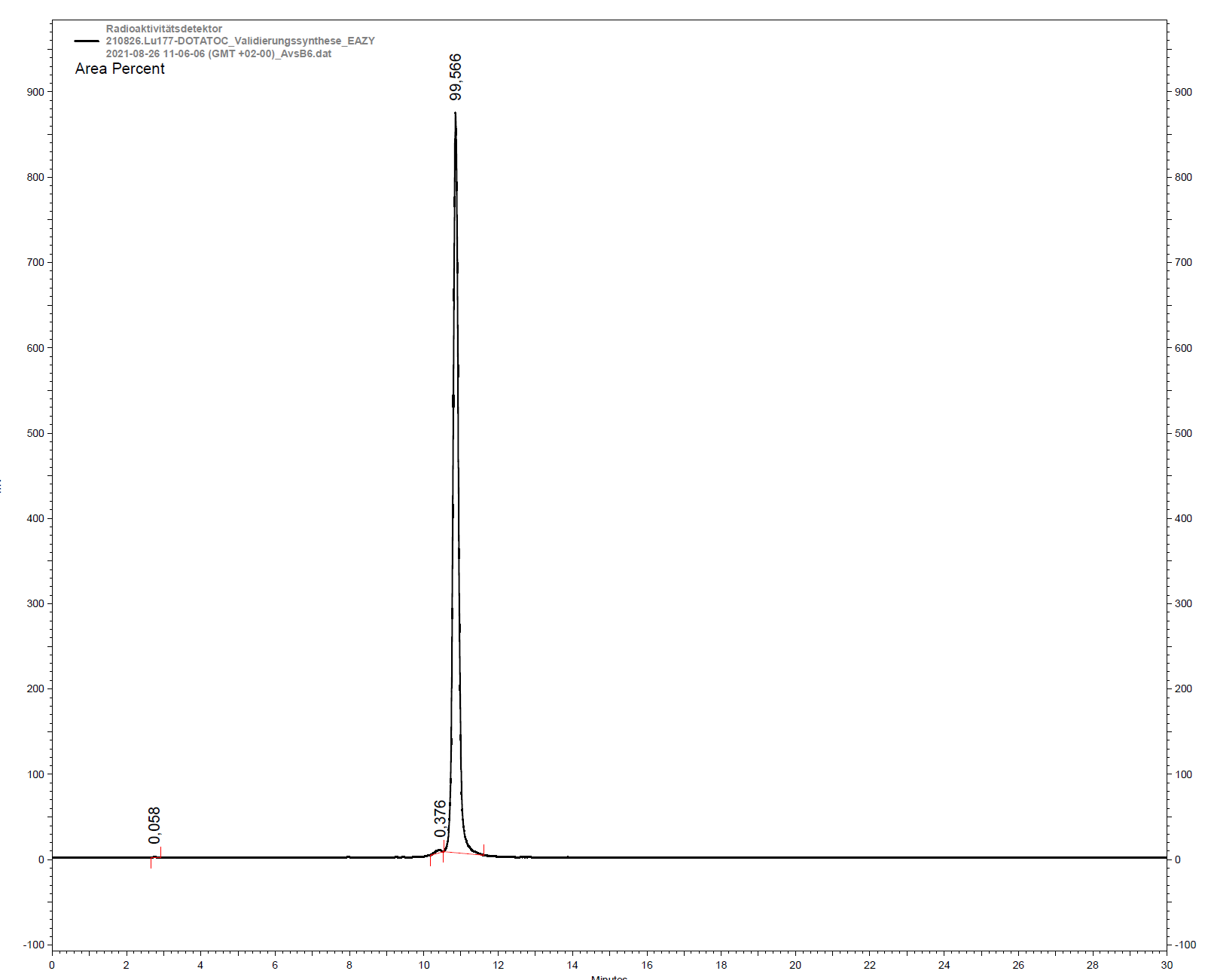


Figure S1. iTLC and radio-HPLC chromatogram of [^177^Lu]Lu-DOTATOC


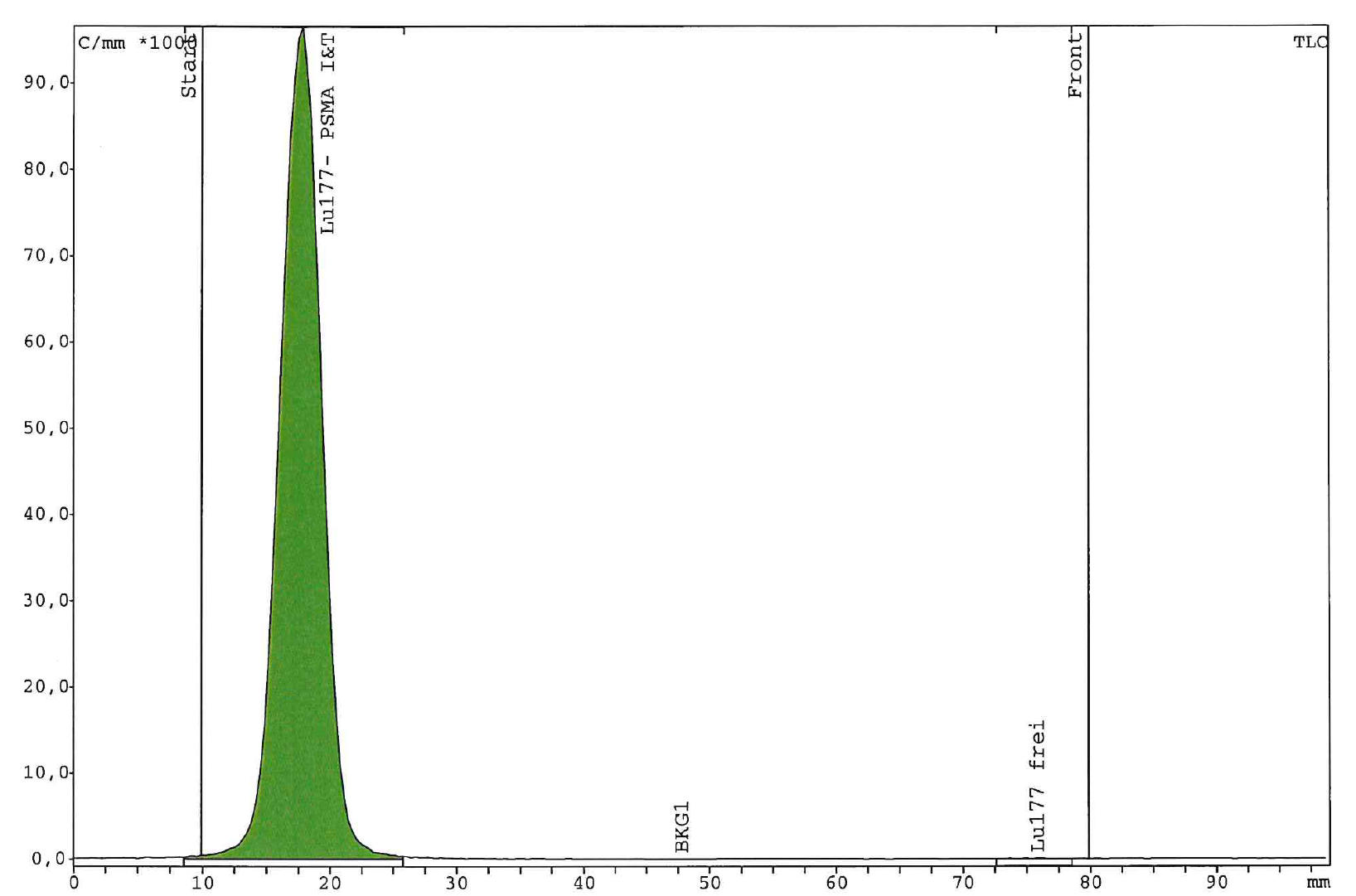

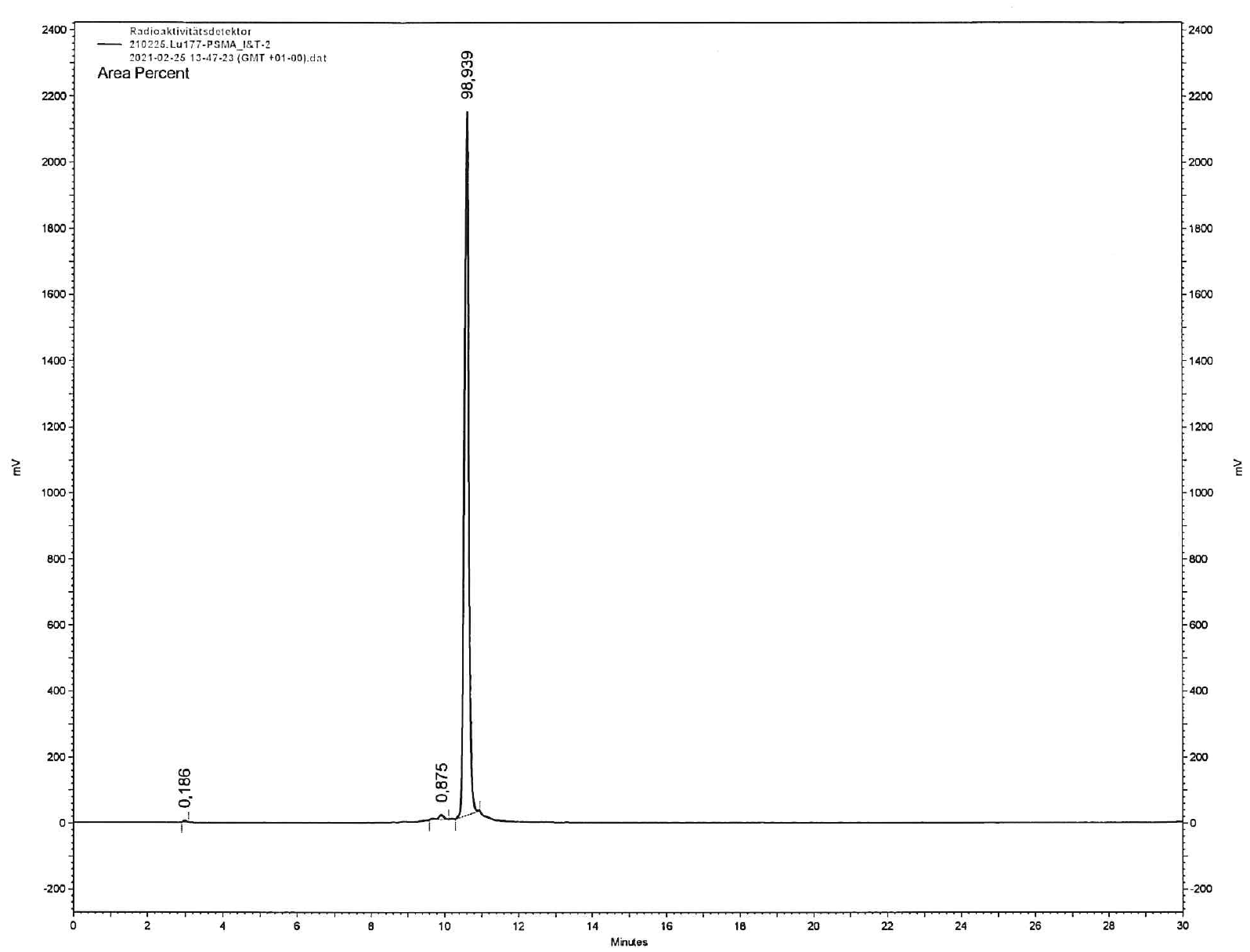


Figure S2 iTLC and radio-HPLC chromatogram of [^177^Lu]Lu-PSMA-I&T


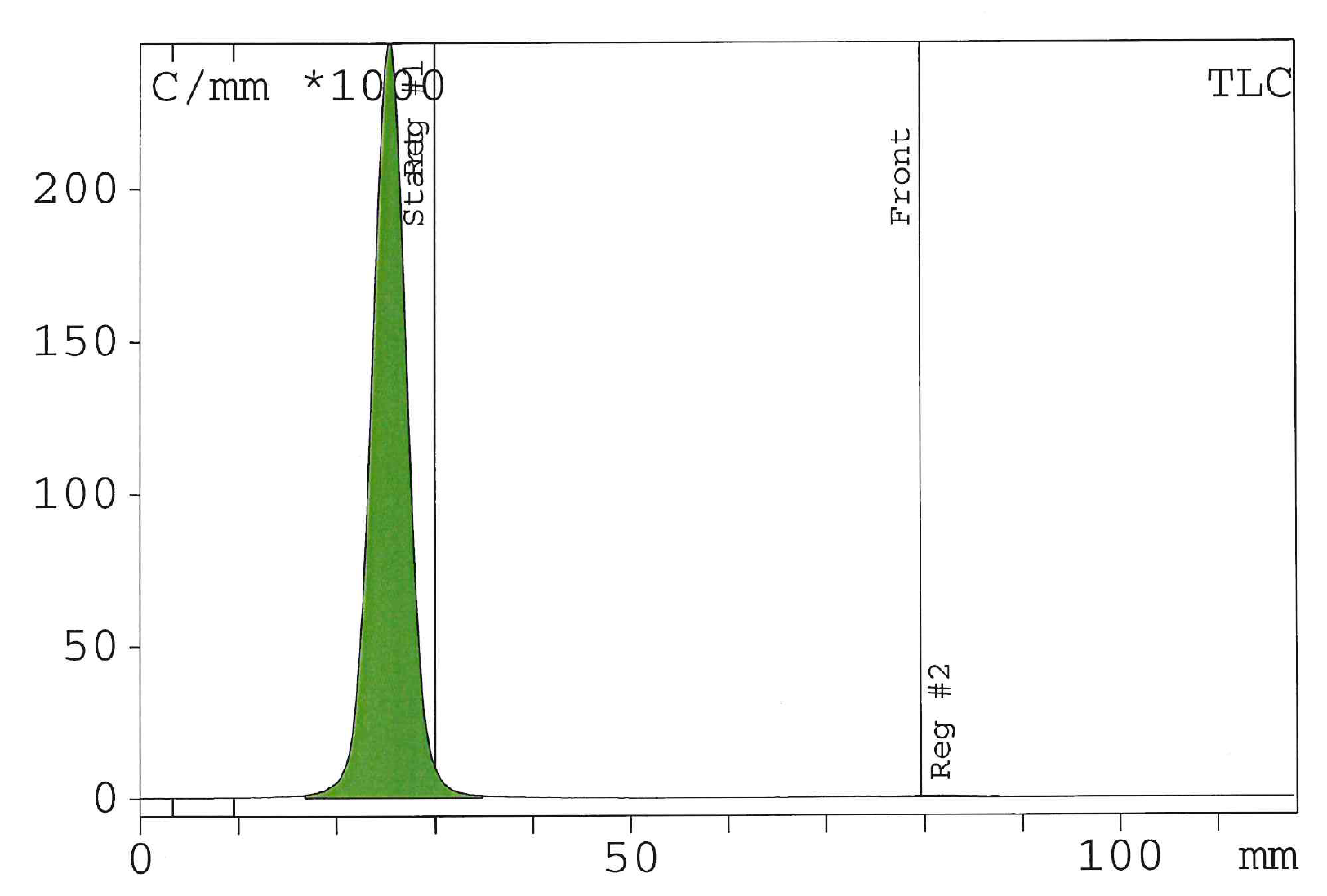

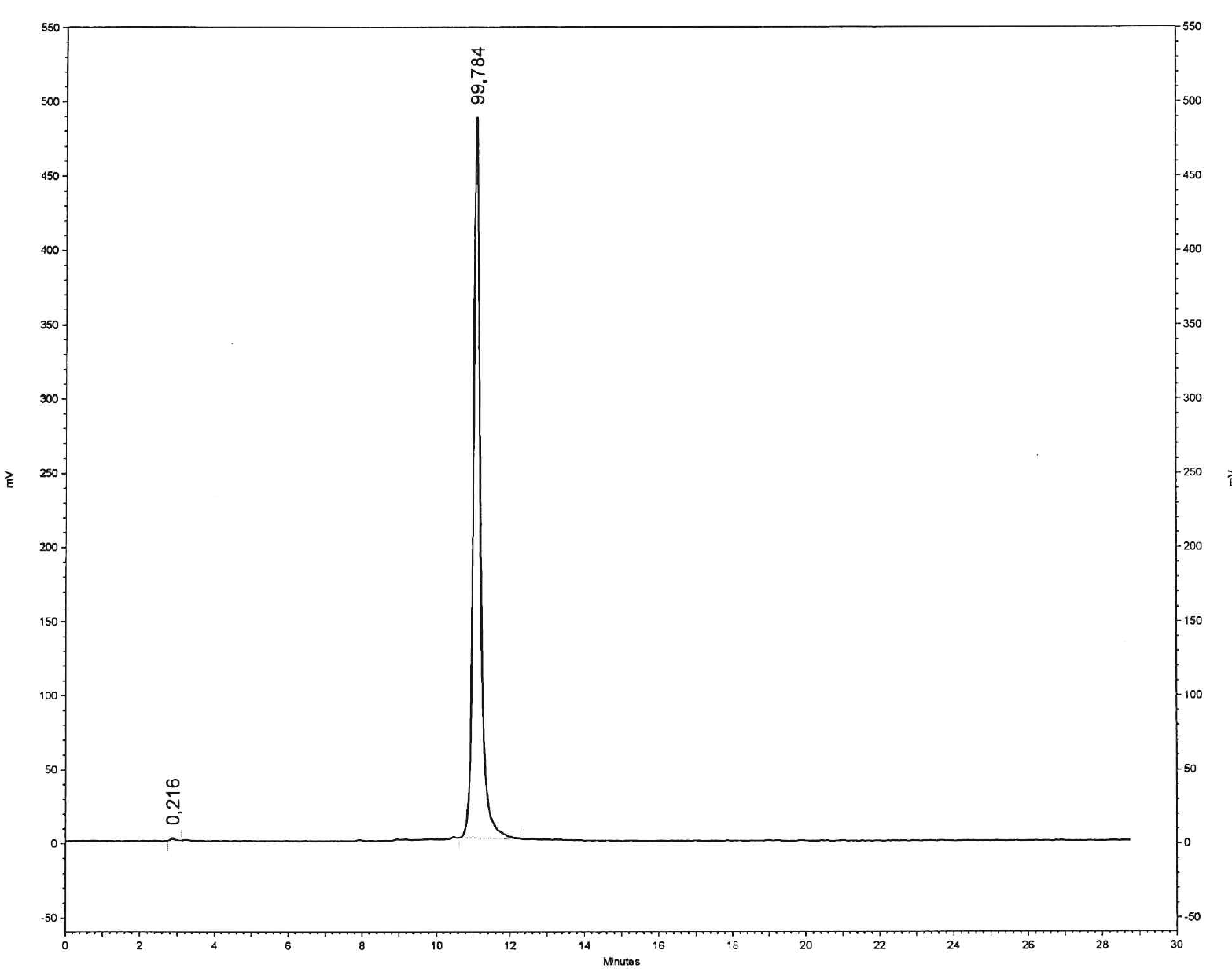


Figure S3 iTLC and radio-HPLC chromatogram of [^177^Lu]Lu-FAPI-46
